# Bolometric detection of Josephson inductance in a highly resistive environment

**DOI:** 10.1038/s41467-023-43668-3

**Published:** 2023-12-01

**Authors:** Diego Subero, Olivier Maillet, Dmitry S. Golubev, George Thomas, Joonas T. Peltonen, Bayan Karimi, Marco Marín-Suárez, Alfredo Levy Yeyati, Rafael Sánchez, Sunghun Park, Jukka P. Pekola

**Affiliations:** 1 PICO Group, QTF Centre of Excellence, Department of Applied Physics, Aalto University School of Science, P.O. Box 13500, 00076 Aalto, Finland; 2 Université Paris-Saclay, CEA, CNRS, SPEC, 91191 Gif-sur-Yvette, France; 3 QTF Centre of Excellence, Department of Physics, Faculty of Science, University of Helsinki, 00014 Helsinki, Finland; 4 Departamento de Física Teórica de la Materia Condensada, Condensed Matter Physics Center (IFIMAC) and Instituto Nicolás Cabrera, Universidad Autonoma de Madrid, 28049 Madrid, Spain

**Keywords:** Superconducting devices, Phase transitions and critical phenomena

## Abstract

The Josephson junction is a building block of quantum circuits. Its behavior, well understood when treated as an isolated entity, is strongly affected by coupling to an electromagnetic environment. In 1983, Schmid predicted that a Josephson junction shunted by a resistance exceeding the resistance quantum *R*_Q_ = *h*/4*e*^2^ ≈ 6.45 kΩ for Cooper pairs would become insulating since the phase fluctuations would destroy the coherent Josephson coupling. However, recent microwave measurements have questioned this interpretation. Here, we insert a small Josephson junction in a Johnson-Nyquist-type setup where it is driven by weak current noise arising from thermal fluctuations. Our heat probe minimally perturbs the junction’s equilibrium, shedding light on features not visible in charge transport. We find that the Josephson critical current completely vanishes in DC charge transport measurement, and the junction demonstrates Coulomb blockade in agreement with the theory. Surprisingly, thermal transport measurements show that the Josephson junction acts as an inductor at high frequencies, unambiguously demonstrating that a supercurrent survives despite the Coulomb blockade observed in DC measurements.

## Abstract (web summary)

The predicted dissipative quantum phase transition in a Josephson junction coupled to resistive environment has been examined in recent experiments. In a heat transport experiment, Subero et al. show that the junction acts as an inductor at high frequencies, while DC charge transport confirms insulating behaviour.

## Introduction

Thermal transport by photons in electrical circuits arises from Johnson-Nyquist noise^1,2^ between two resistive elements at unequal temperatures. The resulting current noise flowing in the ideally lossless circuit linking the two resistors provides an efficient thermalization and energy exchange channel at very low temperatures^3,4^. This current noise can be modulated by adding a suitable tunable dissipationless element in the circuit, which can conveniently be implemented by a magnetically^4–6^ or electrically^7^ controlled Josephson device. So far, both theoretical^8,9^ and experimental approaches^4–7^ of photonic heat transport have only considered on-chip resistors with resistance *R* much smaller than the superconducting resistance quantum *R*_Q_, where the environmental back-action effect on the junctions is weak. More fundamentally, energy transport at different frequencies through quantum coherent systems strongly coupled to a resistive environment, characterized by the dissipation strength *R*_Q_/*R*, remains largely unexplored^10,11^.

It is well-established that the electrical transport properties of a superconducting junction depend on the electromagnetic environment in which it is embedded. Charge transport through a tunnel junction in an ohmic environment with resistance comparable to the resistance quantum is suppressed at low voltage bias and temperature because of Coulomb blockade^12^, and the extension of this phenomenon to superconducting junctions, which are intrinsically phase-coherent, comes naturally^12–15^. Recent experiments using this effect include the production of antibunched photons at high rates^16^ and suppressing the Andreev bound state-induced zero bias anomaly, which could be beneficial in the arduous search for Majorana quasiparticles^17^. For a Josephson junction shunted by a resistor with resistance exceeding *R*_Q_, the supercurrent peak (i.e., current at zero applied voltage bias) is predicted to disappear, being shifted to finite voltage as a result of inelastic Cooper pair tunneling^12^. This is accompanied by a sub-linear current-voltage characteristic as $I \sim {V}^{\frac{2R}{{R}_{{{{{{{{\rm{Q}}}}}}}}}}-1}$ at low bias voltages and extremely low temperature, specifically when *k*_B_*T* ≪ *eV* ≪ *R*_Q_*E*_c_/*Rπ*, with *k*_B_ the Boltzmann constant, *E*_c_ the charging energy, and *e* the elementary charge. According to the theory, a phase transition should occur at *R* = *R*_Q_. This transition, which can be associated with the one predicted by Schmid^18^ and Bulgadaev^19^, was first tested in DC charge transport experiments^13,15,20–22^. However, recent admittance measurements of small junctions, shunted by a highly ohmic environment^23^ called into question the scenario of a dissipative phase transition, leading to further debate about the very existence of this transition^24–29^.

In this context, we present a heat transport experiment in which a small tunable junction (effectively a superconducting quantum interference device SQUID) is embedded in a Johnson-Nyquist setup with hot and cold resistors with resistances *R* > *R*_Q_ to explore this regime. The SQUID geometry enables magnetic-flux control of the photonic heat current^4^. It is intended to demonstrate the destruction or resilience of the Josephson coupling through the observations of heat flow oscillations, or lack thereof if the junctions are truly insulating. We find that the magnitude of heat current flowing from one resistor to another remains close to the value given by the quantum limit, and it exhibits clear oscillations with the external magnetic flux, similar to the systems embedded in a low impedance environment^4^. While this observation might point toward the survival of a supercurrent at high frequencies, a control experiment on DC charge transport (angular frequency *ω* = 0) shows clear suppression of the charge current at low voltage bias caused by the environmental Coulomb blockade, in line with theoretical predictions and previous experimental results^13,20^. This apparent contradiction, highlighting the role of heat transport as a complementary probe when many-body correlations are present^30,31^, is discussed within the existing theoretical and experimental literature.

## Results

### Experimental setup

**Fig. 1:**
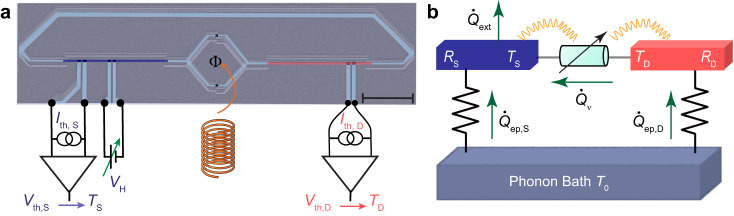
Experimental setup and principle of the photonic heat transport in high ohmic environment. **a** Colored scanning electron micrograph (scale bar: 5 μm) highlighting the (Cr) normal metal (blue and red) and the aluminum superconducting leads (light blue). The Josephson energy of the SQUID is tuned with an external magnetic field. Aluminum leads (vertical, light blue) are connected through an oxide tunnel barrier to the Cr-strip to cool down its electrons locally or as an electronic temperature sensor using a floating DC current source. **b** Schematic illustration of the thermal model of the system. The drain-source heat flow ${\dot{Q}}_{\nu }$ is adjusted by the SQUID. The source and drain electron baths are thermally coupled to the phonon bath (which here is hotter), receiving a power ${\dot{Q}}_{{{{{{{{\rm{ep}}}}}}}},{{{{{{{\rm{S}}}}}}}}}$ and ${\dot{Q}}_{{{{{{{{\rm{ep}}}}}}}},{{{{{{{\rm{D}}}}}}}}}$, respectively. Wiggly lines highlight the strong interaction between the SQUID and the ohmic environment, mediated by photons.

Our device (see Fig. 1a for an SEM image) consists of a SQUID between two nominally identical on-chip thin chromium (Cr) resistors acting as thermal baths, from now on referred to as (hot) drain and (cold) source with resistances denoted by *R*_D_ and *R*_S_, respectively. Each arm of the SQUID is galvanically connected to one source and drain resistor of volume Ω = 10 × 0.1 × 0.014 μm^3^ and whose resistance is nominally equal to that of an independently measured resistor with same dimensions on the same chip, with a value *R*_S_ = *R*_D_ = 11 ± 0.5 kΩ (see Supplementary Note 1). The distance between the SQUID and the resistors is kept short (a few microns) to avoid suppression of environment-induced effects via stray capacitance. The series configuration of the SQUID and resistors is further closed into a loop by a superconducting line. This warrants efficient electromagnetic heat transport through improved impedance matching^32^. The clean contact between chromium and superconducting aluminum leads (see Supplementary Note 2) serves as an Andreev-mirror^33^, which enables essentially perfect conversion to charge transport by Cooper pairs in the superconducting strips while effectively suppressing quasiparticle heat diffusion along them at low temperatures (*T* ≲ 0.2*T*_c_ ~ 260 mK for aluminum)^32^. Four external superconducting leads are contacted with the source resistor through a thin oxide barrier, forming NIS-tunnel junctions. A pair of these junctions is used to measure the electronic temperature (in the case of quasi-equilibrium where the electron temperature is well-defined^34^) by applying a small DC-current bias through it, whereas another pair is used to locally cool the resistor when voltage-biased^34^. The electron temperature of the drain resistor is measured simultaneously by another SINIS junction structure, as depicted in Fig. 1a. We have presented data on two samples, henceforth called Sample I and Sample II.

### DC measurements of the replica sample

**Fig. 2:**
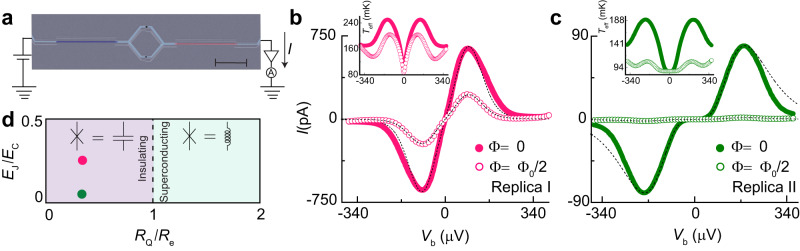
DC charge transport measurements. **a** Scanning electron micrograph of one of the Replica samples (scale bar: 5 μm) with the schematics of the IV measurement. **b**, **c** A close-up of the IVC at the low-bias voltage for the two Replica samples measured at a cryostat temperature of 87 mK exhibiting the Coulomb blockade feature. The measurements were recorded at two magnetic flux values Φ = 0 (solid circles) and Φ = Φ_0_/2 (open circles). The dashed lines in (**b**) and (**c**) are the theoretical results obtained by the standard *P(E)*-theory for two different magnetic flux values. For Replica I in (**b**), the fit parameters are: critical current *I*_C_ ~ 7 nA, Josephson energy *E*_J_ ~ 0.17 K, charging energy *E*_C_ ~ 0.6 K and, for Replica II in (**c**): *I*_C_ ~ 3 nA, *E*_J_ ~ 0.08 K, *E*_C_ = 1.4 K. Cr-strips' resistance is *R*_e_ = 11 kΩ for both samples. The inset in (**b**) and (**c**) show the effective temperature of the resistor at low voltage bias. **d** Illustration of the Schmid phase diagram for a Josephson junction attached to a resistive environment *R* at zero temperature. Here, *R* = *R*_S_ + *R*_D_ = 2*R*_e_ is the total resistance of the environment. Our samples are well placed in the insulating part, represented by the two points.

We first measure the current-voltage characteristics (IVC) of a reference sample (see Fig. 2a) on the same chip, made during the same fabrication run, from now on called “Replica”, with nominally equal parameters as the main sample. This provides estimates of parameters for the heat transport experiments and enables comparison between charge and heat transport behavior. Note that although the geometries of the samples used for these measurements are slightly different, the central part of the two samples (resistors + SQUID) are nominally identical. In the sample used for the heat transport experiment, the superconducting loop keeps the current noise flowing within a closed loop and helps us define the noise power transmission coefficient.

Figure 2b, c shows the IVC for the two Replica samples at a phonon temperature of *T*_0_ = 87 mK in the low bias region at two different magnetic flux values Φ = 0 (solid circles) and Φ = Φ_0_/2 (open circles) with Φ_0_ = *πℏ*/*e* the superconducting magnetic flux quantum. Suppression with respect to the unblocked case is observed in the low-bias DC current through the SQUID, which is more robust for Replica II (panel c) due to its higher charging energy *E*_C_. This observation is well understood in the framework of dynamical Coulomb blockade^12^: the resistive environment impedes charge relaxation after a Cooper-pair tunneling event through junctions with high charging energy, which translates to a conductance reduction at low energy. The value of *E*_C_ (0.6 K for Replica I and 1.4 K for Replica II) is extracted from the current peak feature appearing in the IVC, which for small Josephson junctions in contact with an resistive environment with *R* > *R*_Q_ occurs at a voltage bias *eV*_b_ ~ 2*E*_C_^13,35^. The Josephson energy is estimated by using the Ambegaokar-Baratoff relation as ${E}_{{{{{{{{\rm{J}}}}}}}}}^{AB}={{{\Phi }}}_{0}{{\Delta }}/4e{R}_{{{{{{{{\rm{J}}}}}}}}}$ (0.07 K for Replica I and 0.03 K for Replica II), with Δ ≈ 200 μeV the aluminum superconducting energy gap and *R*_J_ the quasiparticle tunnel resistance of the SQUID. This resistance is obtained experimentally, see Supplementary Note 3 for more details.

The dashed lines in Fig. 2b, c are the theoretical results obtained by the standard *P(E)* theory^12^ under the condition *E*_J_ ≪ *k*_B_*T* in an *RC*-environment, which highlights the effect of the electromagnetic environment on Josephson phase fluctuations (see Supplementary Note 4). Despite the fact that the condition *E*_J_ ≪ *k*_B_*T* is not well satisfied for either of the two samples, one can use a simple rule: if the Josephson energy *E*_J_ is less than Josephson energy obtained from Ambegaokar-Baratoff ${E}_{{{{{{{{\rm{J}}}}}}}}}^{AB}$ (which is our case), then *P*(*E*) theory should still hold regardless of the condition *E*_J_ ≪ *k*_B_*T* and the relatives’ magnitudes of *E*_J_ and *E*_C_^36^. In these calculations, we include overheating caused by the applied bias, as depicted in the inset of Fig. 2b, c, resulting in good agreement with the data at low voltage bias. However, a noticeable discrepancy is observed at high voltages for Replica II. A possible reason for this discrepancy could be that, at voltage beyond *V*_b_ ~ 2*E*_C_/*e*, the Josephson frequency is high, *eV*/*πℏ* > 100 GHz: in this range, the impedance of the environment may significantly differ from our simple RC model. Furthermore, at these voltages closer to the superconducting gap *V* ~ 2Δ/*e* quasiparticles can contribute but are not included in *P(E)* theory. On the other hand, the data at Φ = Φ_0_/2 is fitted by considering an asymmetry factor of the SQUID critical current *d* = 0.58 for Replica I and *d* = 0.15 for Replica II, respectively. Note that the Josephson energy *E*_J_ is a fitting parameter in the model. Finally, Fig. 2d shows the Schmid phase diagram with the two points related to each Replica sample, showing their insulating character as expected.

### Heat transport measurements

**Fig. 3:**
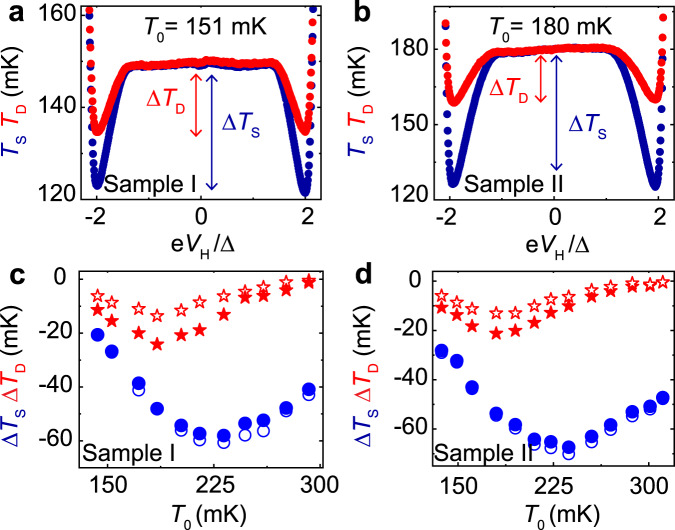
Electronic refrigeration. **a**, **b** Electronic temperature of the source *T*_S_ (blue points) and drain *T*_D_ resistor (red points) at Φ = 0 for samples I and II, respectively, as a function of the heating voltage *V*_H_ applied on the source resistor. The measurements were recorded at phonon temperature *T*_0_ = 151 mK (Sample I) and 180 mK (Sample II). **c**, **d** Temperature drops of the source Δ*T*_S_ (circles) and the drain Δ*T*_D_ (stars) recorded at two magnetic flux values Φ = 0 (solid symbols) and Φ = Φ_0_/2 (open symbols) against *T*_0_. The error bars are not shown since their values are smaller than the symbols.

With these results as a reference, we now turn to heat transport measurements, the main focus of this work. The power flowing from drain to source ${\dot{Q}}_{\nu }$ under a thermal gradient is determined by measuring the electronic temperatures of the drain *T*_D_ and the source *T*_S_. The temperature difference is generated by DC biasing the source resistor with a voltage *V*_H_ ≲ 2Δ/*e* that enables electronic cooling of the source by removal of hot electrons^32,34^. This effect can be observed in Fig. 3a, b, for the two samples at a fixed temperature *T*_0_ in each case. In steady-state, these temperatures involve the different energy relaxation channels in the system, as illustrated in the thermal model that accounts for our setup shown in Fig. 1b. By energy conservation, a direct relation (valid at temperatures *T*/*T*_C_ < 0.2 when quasiparticle heat diffusion along the superconductor is exponentially suppressed^32^), between ${\dot{Q}}_{\nu }$ and the temperatures (*T*_S_, *T*_D_, *T*_0_) measured in the system is found:

1 $${\dot{Q}}_{\nu }({T}_{{{{{{{{\rm{S}}}}}}}}},\,{T}_{{{{{{{{\rm{D}}}}}}}}},\,{{\Phi }})={\dot{Q}}_{{{{{{{{\rm{ep,D}}}}}}}}}({T}_{{{{{{{{\rm{D}}}}}}}}},\,{T}_{0}),$$

where ${\dot{Q}}_{{{{{{{{\rm{ep,D}}}}}}}}}={{\Sigma }}\Omega [{T}_{0}^{5.93}-{T}_{{{{{{{{\rm{D}}}}}}}}}^{5.93}]$ is the electron-phonon heat current governed by the drain resistor. Here, Σ is the electron-phonon coupling constant of the normal metal, which was measured independently to be Σ = (12 ± 0.25) × 10^9^ WK^−5.93^ m^−3^, see Supplementary Note 1 and Supplementary Fig. 1c. With our experimental setup (see Fig. 1a), we have full control of all temperatures and, therefore, the powers involved in the system, leading to an accurate and fully calibrated measurement of the thermal conductance between the drain and source.

Figure 3a, b shows the measured electronic temperature of the source and drain resistor with the applied voltage bias *V*_H_ for each sample at a representative phonon temperature *T*_0_. Notably, both samples show a significant decrease in the source and drain electronic temperature when the voltage approaches ${V}_{{{{{{{{\rm{H}}}}}}}}}^{{{{{{{{\rm{opt}}}}}}}}}\lesssim 2{{\Delta }}/e$, indicating that the cooling power of the SINIS refrigerator reaches its maximum^34^. Figure 3c, d shows the temperature drops ${{\Delta }}{T}_{{{{{{{{\rm{i}}}}}}}}}={T}_{{{{{{{{\rm{i}}}}}}}}}({V}_{{{{{{{{\rm{H}}}}}}}}}^{{{{{{{{\rm{opt}}}}}}}}})-{T}_{{{{{{{{\rm{i}}}}}}}}}({V}_{{{{{{{{\rm{H}}}}}}}}}=0)$, i = S, D at the maximum cooling bias of the SINIS for the two samples at two different magnetic flux values. At *V*_H_ = 0, the electronic temperature equals the phonon temperature *T*_0_. These drops characterize the thermal coupling between the drain and source, i.e., the thermal conductance from drain to source. Source and drain temperature drops differ for the two magnetic fluxes supplied. This difference is more evident in the temperature drops of the drain, where the drop at Φ = Φ_0_/2 is noticeably weaker than at Φ = 0 at low temperatures, indicating that the photonic channel effectively dominates the transport mechanism at lower temperatures^32^. The significant flux-tunability of the remote cooling process is a characteristic feature of SQUID interference^4^, which suggests that the environmental back-action does not destroy the Josephson coupling at zero DC voltage bias. As *T*_0_ increases above 200 mK, the photon thermal coupling gets smaller with respect to electron-phonon coupling.

To quantify the power transferred from the drain to the source resistor through a photon channel, the electronic temperatures measured on the two baths have been converted to heat current ${\dot{Q}}_{\nu }$ by using Eq. (1) and compared with the maximum power that can be transmitted through a single ballistic channel given by ${\dot{Q}}_{{{{{{{{\rm{Q}}}}}}}}}=\frac{\pi {k}_{{{{{{{{\rm{B}}}}}}}}}^{2}}{12\hslash }({T}_{{{{{{{{\rm{D}}}}}}}}}^{2}-{T}_{{{{{{{{\rm{S}}}}}}}}}^{2})$^37^.

**Fig. 4:**
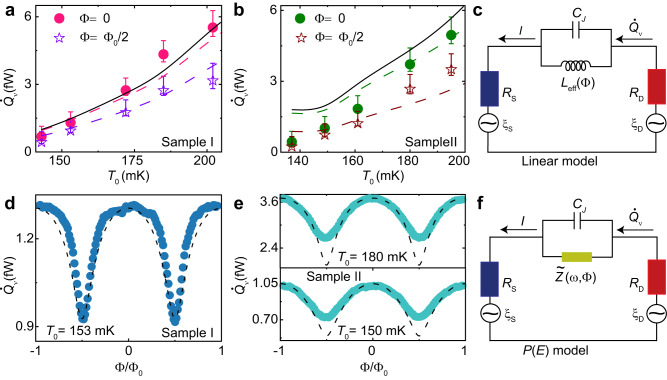
Heat transport mediated by photons and the theoretical model proposed. **a**, **b** Photonic heat current from the drain to the source by using the continuity Eq. (1). The error bars are given in their lower and upper parts, the combination of the thermometer calibration and electron-phonon coupling constant uncertainties, while the upper part also includes the parasitic heat leak on the resistors due to the NIS junctions, with a 0.4 fW upper bound estimate. The solid line represents the power transmitted through a single channel at the quantum limit ${\dot{Q}}_{{{{{{{{\rm{Q}}}}}}}}}$ (see text). The dashed lines are obtained by solving Eq. (2) with a photon transmission probability calculated with the linear circuit model, with the circuit parameters obtained from fitting the IVC of the Replica samples in Fig. 2b, c. **d**, **e** Heat current modulation as a function of the reduced magnetic flux Φ/Φ_0_ through the SQUID at a given temperature *T*_0_. The dashed lines are the application of the linear model (see text), keeping the same circuit parameters as in (**a**) and (**b**). **c** Electric representation of the device in the linear model, where the SQUID is approximated by a re-normalized variable inductor *L*_eff_(Φ) in parallel with the junction geometric capacitor *C*_J_, and **f** *P(E)* model with an effective impedance $\tilde{Z}(\omega,{{\Phi }})$ replacing the Josephson element.

The photonic heat current for the two samples in the temperature range of 140–200 mK is shown in Fig. 4a, b. For Sample I at Φ = 0 (solid circles), the energy is transferred from the drain to the source at a rate very close to that dictated by the quantum of thermal conductance (solid black line). Nevertheless, a minor deviation of ${\dot{Q}}_{\nu }$ from the quantum limit prediction is seen for Sample II. As expected, as soon as the magnetic flux is switched on and reaches the half flux quantum Φ = Φ_0_/2 (star symbols), the heat current ${\dot{Q}}_{\nu }$ for both samples tends to decrease due to weaker photonic coupling^32^.

We then confront the data with theoretical calculations based on the Landauer relation for heat current from drain to source^3^:

2 $${\dot{Q}}_{\nu }=\int\nolimits_{0}^{\infty }\frac{d\omega }{2\pi }\hslash \omega \tau (\omega,{{\Phi }})\left[\frac{1}{{e}^{\hslash \omega /{k}_{{{{{{{{\rm{B}}}}}}}}}{T}_{{{{{{{{\rm{D}}}}}}}}}}-1}-\frac{1}{{e}^{\hslash \omega /{k}_{{{{{{{{\rm{B}}}}}}}}}{T}_{{{{{{{{\rm{S}}}}}}}}}}-1}\right],$$

where *τ*(*ω*, Φ) is the transmission probability of the thermal radiation from the drain to the source at angular frequency *ω*. In the limit of small phase fluctuations around a given average phase bias *φ*, the SQUID can conveniently be approximated by a harmonic oscillator. In electrical terms, this translates to an effective phase-dependent Josephson inductance ${L}_{{{{{{{{\rm{eff}}}}}}}}}({{\Phi }})=\hslash /(2e| {I}_{{{{{{{{\rm{c}}}}}}}}}({{\Phi }})| \langle \cos \varphi \rangle )$ in parallel with a capacitance (see Fig. 4c), where $\langle \cos \varphi \rangle$ is an average over phase fluctuations^26,38^. Within this linear model, and by assuming the lumped approximation (valid since the dominant radiation wavelength *λ*_th_ = *hc*/*k*_B_*T* ~ 10 cm at 150 mK is much larger than the circuit characteristic dimensions ~50 μm), the power transmission coefficient can be explicitly written^3,9^ as *τ*(*ω*, Φ) = 4*R*_S_*R*_D_/∣*Z*_T_(*ω*, Φ)∣^2^ (see “Methods”), with *Z*_T_(*ω*, Φ) the frequency-dependent total series impedance of the circuit. In this framework, the maximum heat transfer is expected for a perfect impedance matching when *R*_S_ = *R*_D_ and when the phase fluctuations are small, i.e., $\langle \cos \varphi \rangle \simeq 1$ under no net electrical bias (see Supplementary Discussion). However, for strong phase fluctuations at high resistances *R*_S_ + *R*_D_ > *R*_Q_, one naively expects the average value of the cosine almost to vanish, $\langle \cos \varphi \rangle \,\approx \,0$. Indeed, we have shown above that the DC charge transport measurements of the Replica are well described by the usual *P*(*E*) theory of Coulomb blockade, which implies $\langle \cos \varphi \rangle \,\approx \,0$. Assuming this, the SQUID can be regarded as a parallel connection of a capacitor *C*_*J*_ and of high effective impedance $\tilde{Z}(\omega,{{\Phi }})\propto 1/{I}_{{{{{{{{\rm{c}}}}}}}}}^{2}({{\Phi }})$, see Fig. 4f. Then the modulation of the Josephson coupling by flux almost does not affect the transmission probability *τ*(*ω*, Φ), and the oscillations of the heat flow are expected to be very small. Below we will show that the strong modulation of the heat flux observed in our experiment is consistent with the assumption of nonvanishing $\langle \cos \varphi \rangle$ rather than with $\langle \cos \varphi \rangle \,\approx \,0$. This finding is similar to that in the recent study conducted by Pechenezhskiy et al.^39^ on a fluxonium qubit shunted by a high impedance environment in which they have revealed the presence of non-vanishing ground state renormalization $\langle 0| \cos \varphi | 0\rangle$ in the diverging inductance limit and the Bloch band structure of a single Josephson junction with *E*_J_ ~ *E*_C_, which leads to modulations in the transition frequency with the external magnetic flux. Our work, combined with theirs, will be useful in developing a general theory that can clarify the dynamics of a Josephson junction at finite frequencies in a high impedance environment.

The dashed lines displayed in Fig. 4a, b are the theoretical results obtained by solving Eq. (2) within the linear model for the corresponding magnetic fluxes applied. For Sample I, reasonable agreement with the experimental data at Φ = 0 is found if we use the bare Josephson junction inductance $L({{\Phi }})=\frac{\hslash }{2e{I}_{{{{{{{{\rm{c}}}}}}}}}({{\Phi }})}$ in the calculated *Z*_T_(*ω*, Φ). Nevertheless, for Sample II, a significant deviation from the data is observed as the temperature is lowered. This deviation can be captured if we set the re-normalization parameter $\langle \cos \varphi \rangle=0.256$. Using the renormalization $\langle \cos \varphi \rangle$ as a free parameter in the fitting is a simple, phenomenological way to emphasize that $\langle \cos \varphi \rangle \,\ne \,0$, which is unexpected for small Josephson junctions in series with a large resistance (*R* ≫ *R*_*Q*_) when considering the DC behavior. Regardless of the value used, these oscillations indicate that the photonic heat from the drain to the source, i.e., current noise over a bandwidth 0 − *k*_B_*T*/*h* ~ 4 GHz, is mainly transmitted through the Josephson inductor channel, which acts as a low-pass filter. At Φ = Φ_0_/2 (dashed purple and dark red lines), the power is reduced as expected, in fair agreement with the data. In this regime, the Josephson critical current is vanishingly small (making the inductance essentially infinite at the relevant frequencies); consequently, the power transmission from the drain to the source takes place mainly through the junction capacitance *C*_J_, which acts as a high-pass filter in the transmission and thus only enables a small fraction of the thermal fluctuations to be transmitted as current in the circuit. The calculation was performed using an asymmetry parameter *d* = 0.15 (which does not coincide with that obtained for replica I, but matches much better the closed SQUID data in heat transport) for sample I and is coincidentally the same for sample II. Furthermore, the heat current calculated within the *P(E)* theory at Φ = 0 shows a decrease of ${\dot{Q}}_{\nu }$ to the level of the power obtained in the linear model at Φ = Φ_0_/2 (see Supplementary Fig. 5b, c).

Figure 4d, e show the heat current modulations measured at given phonon temperatures *T*_0_ for samples I and II, respectively. Clear oscillations with period Φ_0_ are observed. This result unequivocally demonstrates that the inductive response of the junction persists in the presence of strong environmental back-action, in contrast to what is observed for charge transport measurements in the Replica. The data is again compared to the theoretical models proposed. On the one hand, for the two examples presented, the heat current modulations are qualitatively captured with the linear model if we use a value of $\langle \cos \varphi \rangle=0.69$ for sample I and $\langle \cos \varphi \rangle=0.256$ for the modulations at *T*_0_ = 150 mK, and $\langle \cos \varphi \rangle=0.92$ at *T*_0_ = 180 mK in sample II, and keeping the asymmetry parameter same as before. On the other hand, the oscillation amplitude predicted by *P(E)* theory (for which $\langle \cos \varphi \rangle \,\approx \,0$) is much smaller than the amplitudes observed (see Supplementary Fig. 5d, e).

Let us now focus on the discrepancy between charge and heat transport measurements. One obvious difference resides in the relevant frequency range: at zero frequency, *P*(*E*) theory of Coulomb blockade describes incoherent Cooper pair tunneling through the junction, and the transition to an insulating state predicted by Schmid and Bulgadaev is observed as *R* becomes greater than *R*_Q_. On the other hand, heat transport deals with non-zero frequency current fluctuations flowing through the junction at zero net voltage bias. This was considered previously^40^ through an extension of the static version of *P*(*E*) theory to finite frequency transport. The derivation relies on the hypothesis of very weak Josephson coupling, 2*E*_J_ < *k*_B_*T*_S,D_. This condition is not well satisfied for both samples. However, this point can hardly justify our contradicting observations: indeed, the discrepancy is the strongest for sample II (as highlighted by the sharp Coulomb gap observed in DC charge transport, see Fig. 2c), where we are closer to this limit. An alternative, motivated by the microscopic description of the Josephson junction in an arbitrary electromagnetic environment^38^, is the existence of an inductive-like shunt in the junctions’ environment, fundamentally due to the BCS gap, which protects the ground state of the junction from strong phase diffusion. The presence of such a shunt would translate as a finite supercurrent peak, which indeed was reported recently^16^. Its absence in our charge measurement (and previous ones^13,20^) contradicts this interpretation.

Note that recent high-frequency measurements of a small Josephson junction in an engineered high impedance environment have revealed the inelastic nature of the scattering process of a photon off the junction^41^. The conceptual similarity between the setup considered there and ours suggests that the description of the junction as a renormalized inductor^38^, while useful for a basic understanding, is too simplistic because the nonlinearities of the junctions are present due to strong phase fluctuations. Nevertheless, our bolometric technique collects photons at energies over a bandwidth ~ *k*_B_*T*_0_/*ℏ* and, therefore, would not distinguish between several down-converted photons out of an inelastic process or a single elastically scattered photon.

In summary, we have experimentally demonstrated through heat transport measurements that a Josephson junction acts as an inductor even in the presence of a highly resistive environment. Though the interpretation of the dissipative transition can be debated^23,26^, the discrepancy between the heat transport measurements and the control charge transport measurements by us here and in previous works^13,20,42^, cannot be accounted for by the existing theory and calls for further developments, both experimental and theoretical. Our findings are important not only from the fundamental physics point of view but also for future applications such as microbolometers or heat sink designs in quantum circuits. On a practical side, we note that any design aiming at increasing resistances for improved, quantum-limited tunable remote electronic cooling^4,32^ is much less sensitive to back-action effects than initially anticipated.

## Methods

### Device fabrication and measurement

The devices were fabricated on 4-inch silicon substrates covered by 300 nm of Si/SiO_2_ in an electron beam lithography (EBL, Vistec EBPG500 + operating at 100 kV) using a Ge-based hard mask process and the conventional shadow evaporation technique^43^. The silicon wafer was coated with 400 nm layers of poly(methylmethacrylate-methacrylate acid) P(MMA-MAA) resist spun for 1 min at 5500 rpm and baked at 180 °C for 20 min, twice. Then, on top of it 22 nm Ge layer was deposited in an electron-beam evaporator, and right after, ~50 nm thick of PMMA was coated with spun at 2500 rpm for 1 min and baked at 160 °C for 1 min. The devices were patterned on the PMMA layer by using electron beam lithography, and afterward, it was developed using a mixture solution with a concentration of 1:3 of methyl-isobutyl-ketone + isopropanol (MIBK). This pattern is transferred to the Ge mask using reactive ion etching (RIE) with tetrafluoromethane CF_4_ plasma. The undercut in the MMA resist was created by oxygen plasma in the same RIE chamber. The metallic parts were made in three evaporation steps: first, a 20 nm layer of Al is evaporated at an evaporation angle of −22°. Then, static oxidation in-situ with pressure around 3 mbar for 3 min is made. This step defines the superconducting finger used as a thermometer, heater, and branch of the SQUID. In the second step, a 20 nm layer of Al is evaporated at an angle of −7°, forming the SQUID and the clean superconducting contact. Finally, a 14 nm layer of Cr is evaporated at an angle of 24° comprising the thermal bath. The nominal loop area of the SQUID for the two samples was 25 μm^2^. The main difference between them lies in the overlap area of the Josephson junction (JJ), which for Sample I is nominally 130 × 140 nm^2^ and for Sample II is 85 × 85 nm^2^. The resist was lifted-off in acetone at 52 °C. Then, the sample is attached to a sample carrier to be electrically connected to it by Al wire bonds for being measured. The bonded sample is placed on a stage with a double brass enclosure that acts as a radiation shield. It is connected to the mixing chamber of a custom-made plastic dilution refrigerator with a base temperature of ~40 mK. DC signals were applied through cryogenic signal lines filtered with lossy coaxial cables with 0–10 kHz bandwidth connected to the bonded sample through a room-temperature breakout box. In order to sweep the SQUID Josephson energy, a perpendicular magnetic field is supplied by applying DC current to an external superconducting magnet inserted around the vacuum can. All the input signals were applied and read out using programmable sources and multimeters. Amplifying current and voltage output signal was accomplished using a room temperature low noise current amplifier Femto DDPCA-300 and voltage amplifier Femto DLVPA-100-F-D, respectively. The cryostat temperature is controlled by applying a voltage across the heater resistance attached to the mixing chamber. The calibration of thermometers was done by monitoring the voltage drop across the SINIS configuration (current biased *I*_th_ = 15 pA) at zero heating bias voltage while varying the cryostat temperature up to 500 mK^34^.

### Photon transmission coefficient

As mentioned in the main text, the transmission probability of the thermal radiation from the source and drain *τ*(*ω*, Φ) used is Eq. (2) has been calculated within the two models. In the linear model approximation, *τ*(*ω*, Φ) can be written as:

3 $$\tau (\omega,{{\Phi }})=\frac{4{R}_{{{{{{{{\rm{S}}}}}}}}}{R}_{{{{{{{{\rm{D}}}}}}}}}}{| {Z}_{{{{{{{{\rm{T}}}}}}}}}(\omega,{{\Phi }}){| }^{2}},$$

with

4 $${Z}_{{{{{{{{\rm{T}}}}}}}}}(\omega,{{\Phi }})={R}_{{{{{{{{\rm{S}}}}}}}}}+{R}_{{{{{{{{\rm{D}}}}}}}}}+\frac{1}{-i\omega {C}_{{{{{{{{\rm{J}}}}}}}}}+\frac{2\pi {I}_{{{{{{{{\rm{c}}}}}}}}}}{-i\omega {{{\Phi }}}_{0}}\langle \cos \varphi \rangle }.$$

In the charge dominated regime (*E*_C_ ≫ *k*_B_*T*_S,D_ ≫ 2*E*_J_) and taking into account the effect of the environment resistors through *P(E)* function, the transmission probability *τ*(*ω*, Φ) for the system studied reads^10^:

5 $$\tau (\omega,{{\Phi }})=	 4{R}_{{{{{{{{\rm{S}}}}}}}}}{R}_{{{{{{{{\rm{D}}}}}}}}}{\left| {R}_{{{{{{{{\rm{S}}}}}}}}}+{R}_{{{{{{{{\rm{D}}}}}}}}}+\frac{1}{-i\omega {C}_{{{{{{{{\rm{J}}}}}}}}}+{\tilde{Z}}^{-1}(\omega,{{\Phi }})}\right| }^{-2}\\ 	+\frac{{\pi }^{2}{I}_{{{{{{{{\rm{C}}}}}}}}}^{2}}{2{e}^{2}}[{P}_{{{{{{{{\rm{S}}}}}}}}}(\omega )-{P}_{{{{{{{{\rm{S}}}}}}}}}(-\omega )][{P}_{{{{{{{{\rm{D}}}}}}}}}(\omega )-{P}_{{{{{{{{\rm{D}}}}}}}}}(-\omega )],$$

where $\tilde{Z}(\omega,{{\Phi }})$ is the effective frequency-dependent impedance and, the functions *P*_S_(*ω*) and *P*_D_(*ω*) represent the probability of photon absorption in the source and the drain resistors, respectively. These functions are defined as:

6 $${P}_{{{{{{{{\rm{l}}}}}}}}}(\omega )=\int\frac{dt}{2\pi }{e}^{i\omega t}{e}^{-{J}_{{{{{{{{\rm{l}}}}}}}}}(t)},$$

here l = S, D and *J*_l_ is the phase-phase correlation function given by^12^:

7 $${J}_{{{{{{{{\rm{l}}}}}}}}}(\omega )=\frac{4{e}^{2}{R}_{{{{{{{{\rm{l}}}}}}}}}}{\pi \hslash }\int\nolimits_{0}^{\infty }d\omega \frac{\coth \frac{\hslash \omega }{2{k}_{{{{{{{{\rm{B}}}}}}}}}{T}_{{{{{{{{\rm{l}}}}}}}}}}(1-\cos \omega t)+i\sin \omega t}{\omega (1+{\omega }^{2}{({R}_{{{{{{{{\rm{S}}}}}}}}}+{R}_{{{{{{{{\rm{D}}}}}}}}})}^{2}{C}_{{{{{{{{\rm{J}}}}}}}}}^{2})}.$$

The effective impedance $\tilde{Z}(\omega,{{\Phi }})$ is defined as:

8 $$\frac{1}{\tilde{Z}(\omega,{{\Phi }})}=\frac{\pi {I}_{{{{{{{{\rm{c}}}}}}}}}^{2}}{2\hslash \omega }\left[P(\omega )-P(-\omega )-i(P(\omega )+P(-\omega )-2P(0))\tan \frac{\pi ({R}_{{{{{{{{\rm{S}}}}}}}}}+{R}_{{{{{{{{\rm{D}}}}}}}}})}{{R}_{{{{{{{{\rm{Q}}}}}}}}}}\right],$$

where *P*(*ω*) is the *P*-function of the effective environment defined by the convolution of the *P(E)*-function of the two resistors:

9 $$P(\omega )=\int\,d{\omega }^{{\prime} }{P}_{{{{{{{{\rm{S}}}}}}}}}(\omega -{\omega }^{{\prime} }){P}_{{{{{{{{\rm{D}}}}}}}}}({\omega }^{{\prime} }).$$

### Supplementary information

Supplementary Information

Peer Review File

## Data Availability

The findings of this study can be supported with data that is accessible upon request from the corresponding author.
