# Supplementary material for: Bolometric detection of Josephson inductance in a highly resistive environment
Source: Nat Commun. 2023 Dec 1;14:7924. doi: 10.1038/s41467-023-43668-3 (PMC10692220; doi:10.1038/s41467-023-43668-3)
Supplement: Supplementary file 1 — Supplementary Information [file 41467_2023_43668_MOESM1_ESM.pdf]

Supplementary information: Bolometric detection of Josephson inductance in a highly resistive environment

Diego Subero,[1,*] Olivier Maillet,[1,2] Dmitry S. Golubev,[1] George Thomas,[1] Joonas T. Peltonen,[1] Bayan Karimi,[1,3] Marco Marín-Suárez,[1] Alfredo Levy Yeyati,[4] Rafael Sánchez,[4] Sunghun Park,[4] and Jukka P. Pekola[1]

[1] *PICO Group, QTF Centre of Excellence, Department of Applied Physics, Aalto University School of Science, P.O. Box 13500, 0076 Aalto, Finland*

[2] *Université Paris-Saclay, CEA, CNRS, SPEC, 91191 Gif-sur-Yvette, France*

[3] *QTF Centre of Excellence, Department of Physics, Faculty of Science, University of Helsinki, 00014 Helsinki, Finland*

[4] *Departamento de Física Teórica de la Materia Condensada, Condensed Matter Physics Center (IFIMAC) and Instituto Nicolás Cabrera, Universidad Autonoma de Madrid, 28049 Madrid, Spain*

SUPPLEMENTARY NOTE 1

Electron-phonon coupling measurement. In order to measure the electron-phonon coupling constant of chromium, we utilized the hot electron effect under steady-state conditions. The studied system is depicted in Supplementary Fig. 1.a. The Cr- film (red) has nominally the same dimension as those placed in the main and Replica samples and evaporated with the same target. The electron temperature in the film metal is elevated by applying Joule power $P = IV$ while simultaneously measuring its electron temperature, see Supplementary Fig. 1.b. Assuming that the quasiparticle heat flow through the Al contact is negligible at temperatures below approximately 300 mK due to the good thermal insulation of superconducting Al, the dominant cooling mechanism for electrons in the heated film is electron-phonon scattering. Therefore, under steady-state conditions, we obtain

$$IV = I^2 R_\mathrm{e} = \Sigma_n \Omega (T_\mathrm{e}^n - T_0^n), \tag{1}$$

with Σ_n the electron-phonon coupling, Ω the volume of the Cr-strip. Here, Σ and n are fitting parameters at each bath temperature, and their behaviors are shown in Supplementary Fig. 1.c. Additionally, we measure the resistance of the Cr-strip by fitting the IV curve, resulting to be $R_\mathrm{e} = 11$ kΩ.

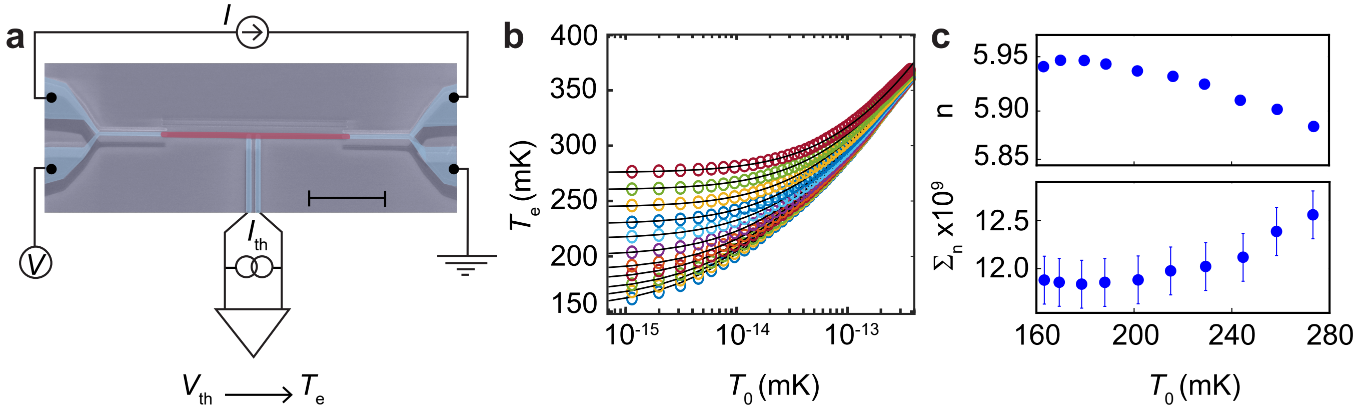

Supplementary Figure 1. **Experimental setup for measuring the electron-phonon coupling.** a.- Colored scanning electron micrograph (scale bar: 5 μm) of chromium (red) used to obtain the electron-phonon coupling, along with a schematic circuit diagram. Vertical aluminum leads are connected to the Cr-strip through an oxide tunnel barrier to monitoring its electronic temperature. b.- Electron temperature of Cr-strip at different T_0 as a function of the applied heating power, with black lines representing fitting results obtained by Supplementary Eq. (1). c.- Temperature dependence of the exponent n and Σ_n (in $\mathrm{WK}^{-n}\mathrm{m}^{-3}$) obtained by fitting measurements to Supplementary Eq. (1).

SUPPLEMENTARY NOTE 2

Cleanliness of the contact between chromium and aluminum. An important assumption for our experiment's overall accuracy is negligible contact resistance between the Cr and Al films, reflecting efficient Andreev reflection as a way to prevent heat diffusion while allowing charge transport at the N/S interface. To confirm the cleanliness of the contact between Al and Cr, we measured various Cr-strips with different lengths but the same nominal transverse dimensions as those stated in Fig. 1a of the main text. The resistance scales linearly with the Cr strip length, as displayed in Supplementary Fig. 2, with an extrapolated residual resistance $\sim 376\ \Omega$ that remains within the typical scatter encountered in the measurements. This value is much smaller than the resistance used in our experiment (~ 11 kΩ), indicating a clean enough interface.

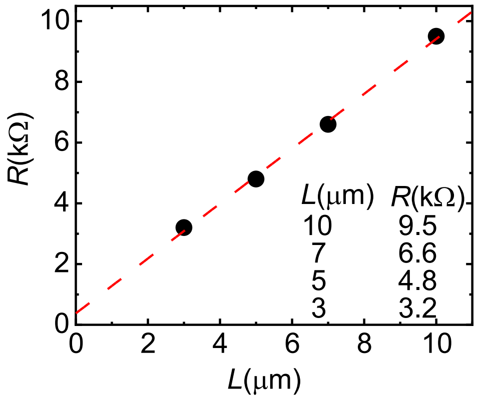

Supplementary Figure 2. **Linear scaling of Cr resistance with the length.** Measured 4-wire resistances of Cr-strips with various lengths. The red line is a linear fit.

SUPPLEMENTARY NOTE 3

Current-voltage characteristic of the Replica sample. The quasiparticle tunnel resistance of the SQUID R_{J} and the superconducting gap were obtained from standard IV measurements as depicted in Supplementary Figs. 3.a and 3.b. The total resistance in series of the Replica $R_{\mathrm{T}} = R_{\mathrm{S}} + R_{\mathrm{D}} + R_{\mathrm{J}}$ with $R_{\mathrm{S}} = R_{\mathrm{D}} = R_{\mathrm{e}}$, was deduced from the slope of the IV-curve at high voltage bias and it is represented by the dashed black line in Supplementary Figs. 3.a and 3.b. These resistances were: 122 kΩ for Replica I and 280 kΩ for Replica II. Hence, the resistance R_{J} was determined by subtracting the effect of series resistance R_{S} and R_{D} from R_{T}. Additionally, the superconducting gap was measured to be $\Delta \simeq 200\ \mu$eV. Thus, the Josephson energy is calculated using the Ambegaokar-Baratoff relation $E_{\mathrm{J}} = \Phi_0 \Delta / 4eR_{\mathrm{J}}$. The single charging energy of the junction $E_{\mathrm{C}} = e^2/2C_{\mathrm{J}}$ is extracted from the current-voltage curve at a low bias, as explained in the main text, from which we estimate the SQUID capacitance to be 1.5 fF for Replica I and 0.7 fF for Replica II.

SUPPLEMENTARY NOTE 4

Josephson current of a small junction embedded in an electromagnetic environment. It is well established that the current-voltage characteristic of a Josephson junction with small critical current embedded in an electromagnetic environment at low voltage bias (shown in Supplementary Fig. 4.a and 4.b), is written as [1, 2]

$$I = \frac{\pi e E_{\mathrm{J}}^2(\Phi)}{\hbar}[P(2eV) - P(-2eV)], \tag{2}$$

where $P(E)$ is the probability function that describes the energy exchange in the inelastic Cooper pair tunneling with the environment. This probability density is given by

$$P(E) = \frac{1}{2\pi\hbar}\int e^{iEt}\langle e^{i\hat{\varphi}(t)} e^{-i\hat{\varphi}(0)}\rangle = \frac{1}{2\pi\hbar}\int dt e^{i\frac{Et}{\hbar} - J(t)}, \tag{3}$$

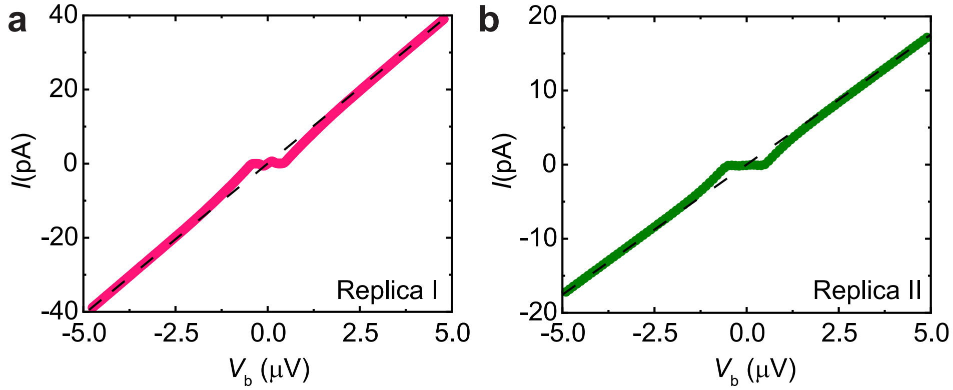

Supplementary Figure 3. **Current-voltage measurements of the replica samples.** a, b.- Current-voltage characteristic at large voltage bias for the two Replica samples. The dashed black line is the linear fit.

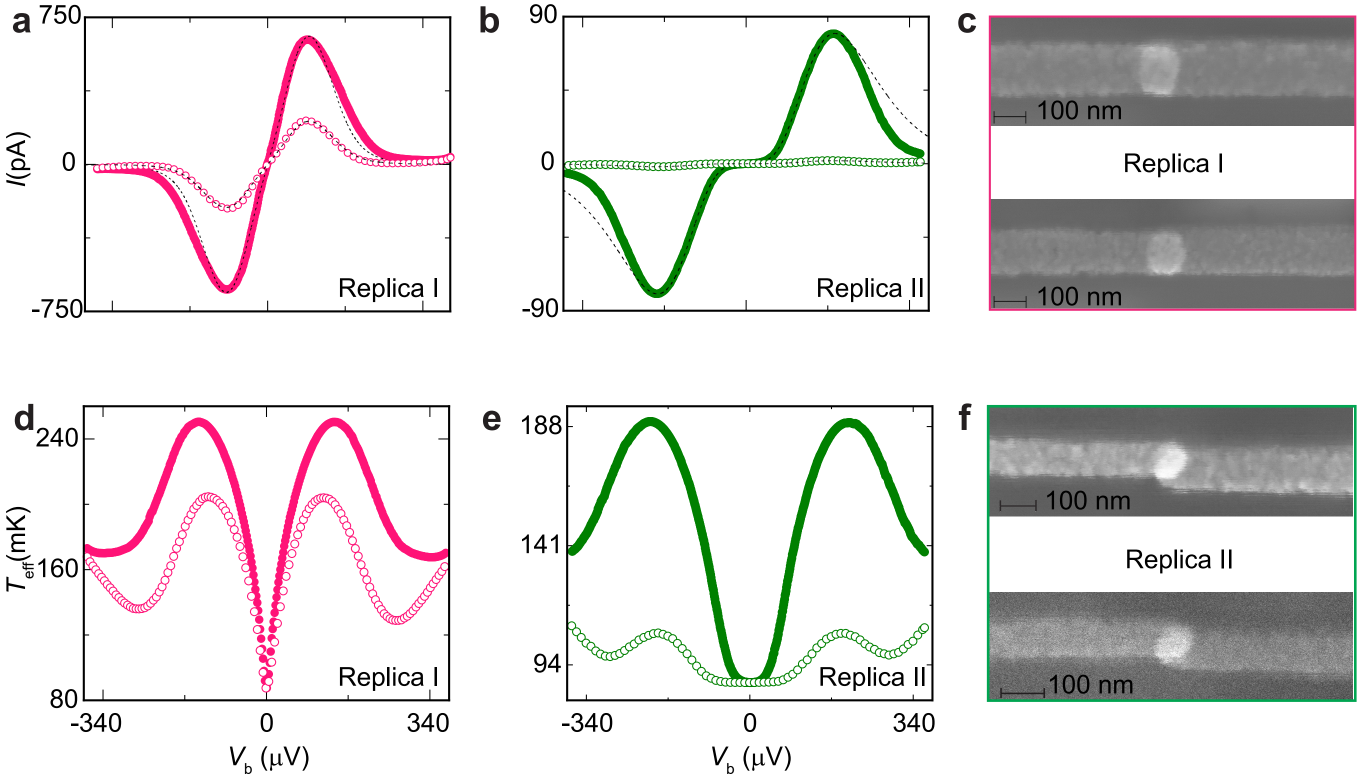

Supplementary Figure 4. **Enlargement of the IV curves shown in Supplementary Fig. 3 in the low voltage bias regime along with SEM images of the Josephson junction**. a, b.- Same data as shown in Fig. 2b and 2c in the main text. The dashed line displays the theoretical results obtained by using Supplementary Eq. (2). d, e.-Bias dependence of the temperature of the resistors for the two Replicas in the low voltage bias regime at two different magnetic fluxes $\Phi = 0$ (solid circles) and $\Phi = \Phi_0/2$ (open circles), obtained from Supplementary Eq. (5). c, f.- SEM images of the two Josephson junctions of the SQUID of the Replicas.

where

$$J(t) = \frac{4e^2}{\pi\hbar}\int_0^\infty d\omega\,\mathrm{Re}[Z_\mathrm{T}(\omega)]\Big[\coth\frac{\hbar\omega}{2k_\mathrm{B}T_\mathrm{eff}(V_\mathrm{b})}\frac{1-\cos\omega t}{\omega} + i\frac{\sin\omega t}{\omega}\Big], \tag{4}$$

is the phase-phase correlation function. Here, $Z_T(\omega)$ and $T_\mathrm{eff}(V)$ are the impedance seen by the junction and the bias voltage-dependent temperature of the resistors. The latter is modeled in the usual way,

$$T_{\rm eff}(V) = \left[T_0^{5.93} + \frac{IV_{\rm b}}{2\Sigma\Omega}\right]^{1/5.93}, \tag{5}$$

and its behavior is shown in Supplementary Figs. 4.d and 4.e. Here, T_0 is the phonon temperature, $\Sigma = 12 \times 10^9$ WK$^{-5.93}$m^{-3} is the electron-phonon constant of the normal metal, and $\Omega = 1.4 \times 10^{-20}$ m^3 is the volume of each resistor. Factor 2 in Supplementary Eq. (5) accounts for the two resistors surrounding the SQUID. The impedance $Z_{\rm T}(\omega)$ is derived from the resistively and capacitively shunted junction (RCSJ) model,

$$Z_T(\omega) = \frac{1}{-i\omega C_{\rm J} + (R_{\rm S} + R_{\rm D})^{-1}}\ ,\ {\rm Re}[Z_T(\omega)] = \frac{R_{\rm S} + R_{\rm D}}{1 + \omega^2 (R_{\rm S} + R_{\rm D})^2 C_{\rm J}^2}\ . \tag{6}$$

Note that the asymmetry of the critical current of the SQUID in Supplementary Eq. (2) is taken into account through the parameter d as [3]

$$E_{\rm J}(\Phi) = E_J(0)|\cos(\pi\Phi/\Phi_0)|\sqrt{1 + d^2\tan^2(\pi\Phi/\Phi_0)}. \tag{7}$$

The SEM pictures displayed in Supplementary Fig. 4.c and Fig. 4.f are used to assess the asymmetry of the SQUID for the two samples that would ideally be symmetric. However, the high peak in the IV curve at $\Phi = \Phi_0/2$ for Replica I (Supplementary Fig. 4.a) suggests us to use a significant asymmetry parameter in fitting, which cannot be justified just based on the SEM pictures.

SUPPLEMENTARY NOTE 5

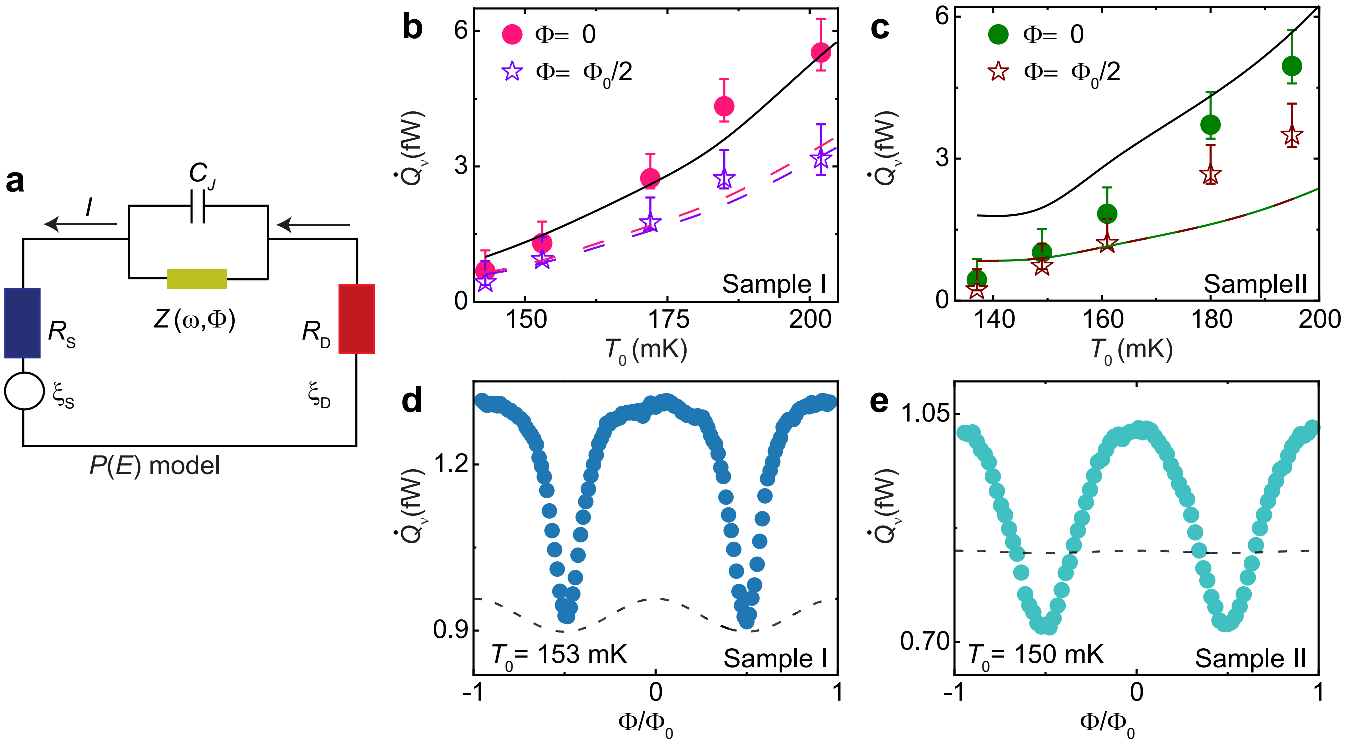

Supplementary Figure 5. $P(E)$**-model for heat transport**. a.-Schematic electric circuit of the device with an effective impedance $\tilde{Z}(\omega,\Phi)$ replacing the SQUID, in series with resistors. b, c, d, e- Same data as shown in Fig. 4 of the main text. The solid line represents the power transmitted through a single channel at the quantum limit $\dot{Q}_{\rm Q}$. The dashed lines are the theoretical results obtained from Supplementary Eq. (8) for the respective magnetic fluxes applied, with the circuit parameters obtained from fitting the IVC of the Replica samples in Fig. 2b and 2c, shown in the main text.

Heat current through a SQUID connected in series with resistors based on the ***P(E)*****-theory**. Here, we consider the system shown in Supplementary Fig. 5.a. The photonic heat flux between the two resistors can be

expressed as (see Ref. [4])

$$\dot{Q}_\nu = \int_0^\infty \frac{d\omega}{2\pi}\,\hbar\omega\,\tau(\omega)\left[\frac{1}{e^{\hbar\omega/k_BT_\mathrm{D}}-1} - \frac{1}{e^{\hbar\omega/k_BT_\mathrm{S}}-1}\right] + \frac{\pi\hbar I_C^2}{4e^2}\int_{-\infty}^{+\infty} d\omega\,\omega\,P_\mathrm{D}(\omega)P_\mathrm{S}(-\omega). \tag{8}$$

This equation is similar to the one deduced by Thomas. *et.al.* [4] for a SQUID connected in parallel with resistors. However, one should re-define the photon transmission probability $\tau(\omega)$ and the functions $P_\mathrm{S}(\omega)$ and $P_\mathrm{D}(\omega)$ for the circuit shown in Supplementary Fig. 5.a. Namely, the transmission probability is expressed as

$$\tau(\omega) = \frac{4R_\mathrm{S}R_\mathrm{D}}{\left|R_\mathrm{S}+R_\mathrm{D}+\frac{1}{-i\omega C_\mathrm{J}+\frac{1}{\tilde{Z}(\omega)}}\right|^2}, \tag{9}$$

where the effective impedance of the junction $\tilde{Z}(\omega)$ was already defined in the methods section of the main text (see Eq. (8) in the main text). To find the expressions for the functions $P_\mathrm{j}(\omega)$ (j= S, D), we write down the classical equation of motion for the Josephson phase in the circuit of Supplementary Fig. 5.a,

$$C\frac{\hbar\ddot{\varphi}}{2e} + \frac{1}{R_\mathrm{S}+R_\mathrm{D}}\frac{\hbar\dot{\varphi}}{2e} + I_C\sin\varphi = \frac{R_\mathrm{S}\xi_\mathrm{S}+R_\mathrm{D}\xi_\mathrm{D}}{R_\mathrm{S}+R_\mathrm{D}}. \tag{10}$$

In order to obtain the equation for the phase, we introduce the new effective resistances $\mathcal{R}_\mathrm{S}$ and $\mathcal{R}_\mathrm{D}$ such that the Supplementary Eq. (10) reads,

$$C_\mathrm{J}\frac{\hbar\ddot{\varphi}}{2e} + \left(\frac{1}{\mathcal{R}_\mathrm{S}}+\frac{1}{\mathcal{R}_\mathrm{D}}\right)\frac{\hbar\dot{\varphi}}{2e} + I_\mathrm{C}(\Phi)\sin\varphi = \eta_\mathrm{S}+\eta_\mathrm{D} \tag{11}$$

where

$$\mathcal{R}_\mathrm{j} = \frac{(R_\mathrm{S}+R_\mathrm{D})^2}{R_\mathrm{j}},\ \eta_\mathrm{j} = \frac{R_\mathrm{j}\xi_\mathrm{j}}{R_\mathrm{S}+R_\mathrm{D}},\quad |\eta_\mathrm{j}|^2_\omega = \frac{R_\mathrm{j}}{(R_\mathrm{S}+R_\mathrm{D})^2}\omega\coth\frac{\hbar\omega}{2k_BT_\mathrm{j}}. \tag{12}$$

Thus, we find the functions $P_\mathrm{j}(\omega)$ by replacing the expression (12) in the Eqs. (3, 4)

$$P_\mathrm{j}(\omega) = \int\frac{dt}{2\pi}\,e^{i\omega t}\,e^{-J_\mathrm{j}(t)}, \tag{13}$$

where

$$\begin{aligned} J_j(t) &= \frac{4e^2}{\pi\hbar}\int_0^\infty d\omega\,\frac{\coth\frac{\hbar\omega}{2k_\mathrm{B}T_\mathrm{j}}(1-\cos\omega t)+i\sin\omega t}{\mathcal{R}_\mathrm{j}\left|-i\omega C_\mathrm{J}+\frac{1}{\mathcal{R}_\mathrm{S}}+\frac{1}{\mathcal{R}_\mathrm{D}}\right|^2} = \frac{4e^2}{\pi\hbar}\int_0^\infty d\omega\,\frac{R_j\left[\coth\frac{\hbar\omega}{2k_\mathrm{B}T_\mathrm{j}}(1-\cos\omega t)+i\sin\omega t\right]}{(R_\mathrm{S}+R_\mathrm{D})^2\left|-i\omega C+\frac{R_\mathrm{S}}{(R_\mathrm{S}+R_\mathrm{D})^2}+\frac{R_\mathrm{D}}{(R_\mathrm{S}+R_\mathrm{D})^2}\right|^2} \\ &= \frac{4e^2}{\pi\hbar}\int_0^\infty d\omega\,\frac{R_\mathrm{j}\left[\coth\frac{\hbar\omega}{2k_\mathrm{B}T_\mathrm{j}}(1-\cos\omega t)+i\sin\omega t\right]}{(R_\mathrm{S}+R_\mathrm{D})^2\left|-i\omega C_\mathrm{J}+\frac{1}{R_\mathrm{S}+R_\mathrm{D}}\right|^2} = \frac{4e^2R_\mathrm{j}}{\pi\hbar}\int_0^\infty d\omega\,\frac{\coth\frac{\hbar\omega}{2k_\mathrm{B}T_\mathrm{j}}(1-\cos\omega t)+i\sin\omega t}{|1-i\omega(R_\mathrm{S}+R_\mathrm{D})C_\mathrm{J}|^2}. \end{aligned} \tag{14}$$

SUPPLEMENTARY DISCUSSION

Impedance matching in the circuit. To better understand the meaning of "improved impedance matching" in our context, let us examine the net power flow between the two resistors described by Eq. (2) in the main text and repeated below,

$$\dot{Q}_\nu = \int_0^\infty \frac{d\omega}{2\pi}\hbar\omega\tau(\omega,\Phi)\left[\frac{1}{e^{\frac{\hbar\omega}{k_BT_D}}-1} - \frac{1}{e^{\frac{\hbar\omega}{k_BT_S}}-1}\right], \tag{15}$$

where $\tau(\omega,\Phi)$ is the transmission coefficient for the power per unit frequency bandwidth from the drain resistor to the source resistor. This generic formula can be obtained with many approaches [5–7]. In a circuit description [5],

which in our case is a simple lumped element one (see main text), $\tau(\omega,\Phi)=\frac{4R_SR_D}{|Z_{\mathrm{T}}(\omega,\Phi)|^2}$, where $Z_{\mathrm{T}}(\omega,\Phi)$ is the total impedance of the loop circuit. This can be expressed as,

$$Z_{\mathrm{T}}(\omega,\Phi)=R_{\mathrm{S}}+R_{\mathrm{D}}+Z_{\mathrm{J}}(\omega,\Phi)+Z_{\mathrm{K}}(\omega). \tag{16}$$

Here, $Z_{\mathrm{J}}(\omega,\Phi)$ characterizes the effective impedance of the SQUID, while Z_{K} accounts for the kinetic impedance of the superconducting loop wire. Consequently, the state of matching is achieved when $Z_{\mathrm{K}}=Z_{\mathrm{J}}=0$, while the resistors possess identical values, i.e., $R_D=R_S$. In practical terms, this means that the equal resistors are interconnected via lossless and negligible inductance lines. In this scenario, $\tau(\omega)=1$ over the whole frequency range, and power is fully transmitted at the quantum limit of thermal conductance $\dot{Q}_\nu=\frac{\pi k_{\mathrm{B}}^2}{12\hbar}(T_{\mathrm{D}}^2-T_{\mathrm{S}}^2)$ [8]. However, it is plausible to consider that the SQUID impedance does not inherently lead to $Z_J=0$, akin to the insights in [9], thereby resulting in an unfavorable matching condition. Therefore, in our design, $Z_{\mathrm{K}}(\omega)\approx 0$ for $\omega\lesssim k_BT/\hbar$. Hence, any deviation from the quantum limit of heat transfer can be attributed to the physics occurring at the SQUID.

* diego.suberorengel@aalto.fi

[1] G.-L. Ingold and Y. V. Nazarov, Charge tunneling rates in ultrasmall junctions, in *Single charge tunneling* (Springer, 1992) pp. 21–107.

[2] O.-P. Saira, M. Zgirski, K. L. Viisanen, D. S. Golubev, and J. P. Pekola, Dispersive thermometry with a Josephson junction coupled to a resonator, Phys. Rev. Applied **6**, 024005 (2016).

[3] A. Ronzani, B. Karimi, J. Senior, Y.-C. Chang, J. T. Peltonen, C. Chen, and J. P. Pekola, Tunable photonic heat transport in a quantum heat valve, Nat. Phys **14**, 991 (2018).

[4] G. Thomas, J. P. Pekola, and D. S. Golubev, Photonic heat transport across a Josephson junction, Phys. Rev. B **100**, 094508 (2019).

[5] D. Schmidt, R. Schoelkopf, and A. Cleland, Photon-mediated thermal relaxation of electrons in nanostructures, Phys.Rev. Lett **93**, 045901 (2004).

[6] T. Ojanen and A.-P. Jauho, Mesoscopic photon heat transistor, Phys.Rev. Lett **100**, 155902 (2008).

[7] L. Pascal, H. Courtois, and F. W. Hekking, Circuit approach to photonic heat transport, Phys. Rev. B **83**, 125113 (2011).

[8] A. V. Timofeev, M. Helle, M. Meschke, M. Möttönen, and J. P. Pekola, Electronic refrigeration at the quantum limit, Phys.Rev. Lett **102**, 200801 (2009).

[9] M. Meschke, W. Guichard, and J. P. Pekola, Single-mode heat conduction by photons, Nature **444**, 187 (2006).
